# A machine learning approach to analyse ozone concentration in metropolitan area of Lima, Peru

**DOI:** 10.1038/s41598-022-26575-3

**Published:** 2022-12-21

**Authors:** Natalí Carbo-Bustinza, Marisol Belmonte, Vasti Jimenez, Paula Montalban, Magiory Rivera, Fredi Gutiérrez Martínez, Mohamed Mehdi Hadi Mohamed, Alex Rubén Huamán De La Cruz, Kleyton da Costa, Javier Linkolk López-Gonzales

**Affiliations:** 1 Doctorado Interdisciplinario en Ciencias Ambientales, Universidad de Playa Ancha, Valparaíso, Chile; 2 Laboratorio de Biotecnología, Medio Ambiente e Ingeniería (LABMAI), Facultad de Ingeniería, Universidad de Playa Ancha, Avda. Leopoldo Carvallo 270, Valparaíso, Chile; 3 HUB-Ambiental, Universidad de Playa Ancha, Avda. Leopoldo Carvallo 270, Valparaíso, Chile; 4 Facultad de Ingeniería y Arquitectura, Universidad Peruana Unión, Lima, Peru; 5 Vicerrectorado de Investigación, Universidad Peruana Los Andes, Huancayo, Peru; 6 E.P. de Ingenieria Ambiental, Universidad Nacional Intercultural de la Selva Central Juan Santos Atahualpa, La Merced, Peru; 7 Holistic AI, London, UK

**Keywords:** Environmental sciences, Environmental impact

## Abstract

The main objective of this study is to model the concentration of ozone in the winter season on air quality through machine learning algorithms, detecting its impact on population health. The study area involves four monitoring stations: *Ate*, *San Borja*, *Santa Anita* and *Campo de Marte*, all located in Metropolitan Lima during the years 2017, 2018 and 2019. Exploratory, correlational and predictive approaches are presented. The exploratory results showed that ATE is the station with the highest prevalence of ozone pollution. Likewise, in an hourly scale analysis, the pollution peaks were reported at 00:00 and 14:00. Finally, the machine learning models that showed the best predictive capacity for adjusting the ozone concentration were the linear regression and support vector machine.

## Introduction

Currently, air pollution is one of the most harmful environmental problems at the local, regional and global levels. Its impacts go beyond ecosystems, harming human health, the economy and environmental sustainability^1^. Most of the world’s population lives in a polluted environment. Although physical activities release different pollutants, the main source of pollution is anthropogenic activities, which accidentally release dangerous chemicals^1,2^. Elevated tropospheric ozone (O$_{3}$) concentrations signal a serious threat to the climate and the environment. In addition, due to industrial processes and urbanization, climate change intervenes in the dispersion of O$_{3}$^2^. Nitrogen dioxide (NO$_{2}$), O$_{3}$, aerosol absorption index (AAI), and carbon monoxide (CO) are key indicators of air pollution. The creation of NO$_{2}$ influences the formation of ozone, through a complex set of reactions with oxygen and free radicals generated from volatile organic compounds (VOC) in the presence of sunlight^3^, which is why the levels highest ozone levels are recorded during periods of sunny weather^4^. On the other hand, chemical ozone loss due to anthropogenic halogens is temperature driven, with greater loss occurring during cold winters, and this pollutant is readily soluble in water, indicating that the presence of precipitation increases the speed at which it dissolves, and in the winter season ozone concentrations decrease^5^. O$_3$ is considered a secondary pollutant, because it results from a photochemical reaction of CO and VOC in the presence of nitrogen oxides (NO$_{x}$ = NO + NO$_{2}$), which allows its high concentrations, developed by emissions of NO$_{x}$ coming from combustion sources^6^. However, for ozone to accumulate to levels harmful to health, there must be continuous recycling between NO and NO$_{2}$. That is why predicting and understanding the rate of formation and emission of ozone is essential both to alert the public about the appropriate intervention, and to evaluate immediate actions on climate behavior^7^.

At the global level, China is one of the countries that presents the most problems with ozone concentrations and emissions^7,8^, since the critical days of O$_3$ pollution are 93 to 575% higher than those of other industrialized countries, with Beijing and Shanghai being the cities with the highest air pollution in recent years^8^. On the other hand, the global ozone load is perceptible to the variation of emissions in tropical and subtropical regions, since in these there are favorable parameters such as high temperatures, intense sunlight and convection, for the ozone production and accumulation, showing the close relationship between climatic variables and O$_3$ concentrations^9^. As a counterpart, some places in the United States and southern Canada have minimal ozone exposure, even being considered “clean places”^10^. In Europe and North America, projects are being carried out to improve air quality, taking into account environmental and climatic factors for greater application^11^. At the same time, seeing the focus on Latin America, it is known that there is a higher exposure rate in areas located near land routes with a high level of vehicular congestion, as well as industrial regions, due to the secondary pollutants that are formed downwind, as in the case of ozone, which is one of the most dangerous pollutants in existence^12^. In recent years, they began to propose and implement measures to improve air quality, with Chile and Brazil being the leaders in terms of change. However, despite this, a study revealed that only 17 countries in Latin America and the Caribbean have regulations and policies regarding ozone as a pollutant^13^. Peru is in the ranking of the countries with the highest rates of air pollution. However, the National Institute of Statistics and Informatics indicates that, at the urban national level, more than half of the population considers that the air in their area is polluted^14^. This situation is associated with the rapid economic and industrial development of Peru, which means the release of pollutants and gases that alter air quality. Almost a third of the total population of Peru resides in Lima, which is why the largest amount of air pollutants are present in the country’s capital city, making Lima one of the thirty most polluted cities in South America^15^. In metropolitan Lima, there is an Automatic Air Quality Monitoring Network System (RAMCA), which is based on low-cost alternative methods. This system has around ten stations, which record atmospheric gases on an hourly basis, among them are: Ate (ATE—East Lima), San Borja (SB—South Central Lima), Campo de Marte (CDM—Lima Central) and Santa Anita (STA—East Lima), currently monitored by the *Servicio Nacional de Meteorología e Hidrología del Perú* (SENAMHI) under the command of the Ministry of the Environment^16^. Lima’s air quality is greatly affected by persistent weather and climate patterns^17^. According to the environmental quality standard for air, it sets levels of concentrations of physical, chemical and biological parameters present in the atmosphere, thus indicating the value allowed for ozone with 100 $\upmu$g/m$^3$ in a period of 8 h^18^.

For his part, the anthropogenic causes such as the burning of fossil fuels in the industrial sector, the high rate of vehicular transport, waste burning and excessive agriculture, excessively alter the levels of greenhouse gases and generate particulate matter, causing an imbalance that affects both the natural ecosystem and the health of human beings^19^. Likewise, the climatological variables such as temperature, wind speed and relative humidity are a fundamental part of the atmospheric system, which influences the spread, increase and accumulation of the pollutant^16,17,19^. Therefore, conceiving seasonal changes, climatic alterations, and potential causes in the area, allow a better monitoring and mitigation plan for pollutants^20^. On the other hand, when examining the correlation between ozone and climatic variables, obtain a greater guide to analyze the periods and critical points of concentration of the pollutant^21^. In this context, understanding air pollutants over a range of space and time is essential for a meaningful assessment of the relationship between air pollutant concentrations and adverse human health effects. However, meteorological variables have a great influence on air pollution through multiple pathways of pollutants^22^. Using statistical and deterministic models, the concentration of pollutants in the air can be addressed. For its part, machine learning facilitates the understanding of air pollution data based on the exposure of the data relationship and the prediction of results, independent of empirical models^23^. It addresses the nonlinearity problem and improves the predictive performance of the model^24^. In Peru, modeling studies for ozone have not yet been carried out. Attempts to take advantage of the high predictive capabilities of machine learning algorithms for modeling are limited. In this sense, our contributions are summarized below:

- We apply machine learning techniques to model the concentration of ozone on air quality in four monitoring stations in Metropolitan Lima during the winter season. These were: ATE, SB, CDM and STA.
- We investigated the climatic and geographic diversity of all monitoring stations, using data collected from three consecutive years (2017, 2018 and 2019).
- The analysis based on machine learning algorithms effectively predicted the ozone concentration on an hourly scale.
- In recent years, air pollution has increased in the capital of Peru, a determining reason for focusing the study on this area, considering that the accelerated automobile and industrial growth are the main causes of pollution.

The rest of the paper is structured as follows: “Materials and methods” that describes the methodology developed based on statistical modeling approaches. Then, “Results” and “Discussion” that presents the main findings of this research compared to other studies. Finally, “Conclusions” that provides the main conclusions, together with some recommendations for future research.

## Material and methods

**Figure 1:**
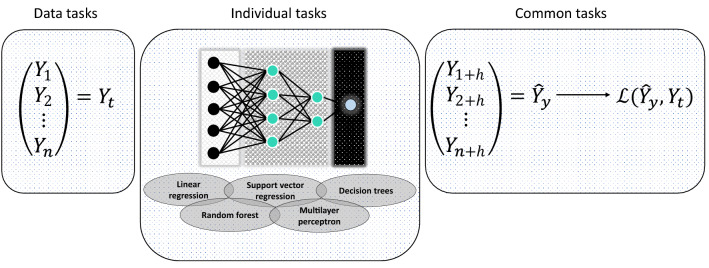
Architecture machine learning: Linear regression, support vector regression, decision trees, random forest, and multilayer perceptron. *Data tasks:* the data related to the ozone concentration in the winter season of 2017, 2018 and 2019 are organized. *Individual tasks:* machine learning models are applied to data that was previously organized. *Common tasks:* the prediction is made and the errors of each model are calculated using performance metrics.

The methodology of this study carried out a data pre-processing. The database was ordered, classified and analyzed for each monitoring station, taking into account the winter period of the city, which runs from June 21 to September 22, both for climatic variables and for ozone. Four monitoring stations located at strategic points in Metropolitan Lima were considered, from 2017 to 2019. It is worth mentioning that the number of monitoring stations in Metropolitan Lima is ten, however, four were selected due to lack of data in the registers. The hourly concentrations of O$_3$ were measured using Teledyne analyzers. The analyser operation includes zero and span verifications, calibrations and detection of leaks. The data are transmitted by telemetry to SENAMHI to be validated after correcting null entries, duplicates, and/or anomalies. Likewise, SENAMHI has a systematic network of conventional and automatic stations that monitor and report the variables under study to a processing center. These stations use high-quality instruments and sensors to measure temperature, relative humidity, wind speed and direction on an hourly scale. In addition, the imputation algorithm called Multiple Imputation by Chained Equations was applied. This algorithm is based on Fully Conditional Specification, where each incomplete variable is imputed by a separate model^25^. This performs multiple imputation to replace missing values in a data set, in this case, for hourly scale records (see Table 1). Likewise, reports were generated in the R Studio and Jupiter notebook programs, to present the descriptive, exploratory, correlational and predictive analyses. The latter was addressed using different machine learning algorithms (Fig. 1) and evaluating its ability to adjust through performance metrics.

**Table 1:** Percentage of imputation for each monitoring station during the years 2017, 2018 and 2019. This analysis is based on the 2256 observations obtained from the winter season.

Year	Monitoring station	Total	Completed	Imputed	% imputed
2017	ATE	2256	2212	44	1.95 %
	CDM	2256	2201	55	2.95 %
	SB	2256	2203	53	2.35%
	STA	2256	2196	60	2.66%
2018	ATE	2256	2216	40	1.77%
	CDM	2256	2221	35	1.55%
	SB	2256	2203	53	2.35%
	STA	2256	2213	43	1.91%
2019	ATE	2256	2226	30	1.33%
	CDM	2256	2212	44	1.95%
	SB	2256	2208	48	2.13%
	STA	2256	2204	52	2.30%

### Study area and monitoring stations

This study focuses on two districts in East Lima and two in Central Lima. The metropolis is characterized by having a temperate climate, with a high and constant atmospheric humidity in winter, despite being considered one of the second driest cities on the planet and this due to the minimum rainfall that it presents near the 9 mm^26^. On the other hand, relative humidity is above 80% throughout the year. Normally, it does not record lower amounts and the speed of the wind coming from the south ranges between 4 and 5 m/s^26^. The air quality in the city is poor, which prevents clean air and good health in the population. The quality varies in time intervals, by hours or minutes^27^. The pollutants move in the city according to the prevailing wind regime. However, the tropospheric ozone is one of the most harmful pollutants that harm human health, that is why it is designated “bad ozone”^28^. But in the metropolitan area it does not exceed the level recommended by Peruvian laws^16^, specifically in the winter season, despite the fact that the general levels are not high compared to those of spring-summer. Ozone is likely to have an impact, even at low concentrations^29^.

For his part, the monitoring stations are located at key points of industrial development and vehicular traffic^30^. The first station is located in the ATE district, which is one of the areas where there is more particulate matter, since it is on both sides of the central expressway and where vehicle traffic has increased^31^. This same phenomenon occurs in the STA district. On the other hand, the SB monitoring station is located in a heavy vehicle traffic zone where excess pollutants are concentrated^32^. Finally, the CDM monitoring station, which is exposed to the frequent emissions of the vehicle fleet and the anthropogenic activities of the place^32^.

### Machine learning modelling

Machine learning is an approach based computational study for deriving knowledge from data. Likewise, trains algorithms to accept and predict new data using statistical analysis. For this study, the monitoring stations were divided into two: training and testing. Five machine learning models, linear regression, random forest, support vector regression, decision trees regression and multilayer perceptron, were used to predict the ozone’s hourly concentration. The model is used to ascertain the independent variables’ potential (meteorological variables) to predict the dependent variable ($\textrm{O}_{3}$ concentration). The model was developed using scikit-learn within the python programming environment. $80\%$ of the dataset was used for model training and the rest of the dataset was used to test the model. Model validation was done using the coefficient of the determinant $\left( \textrm{R}^{2}\right)$, which tests for models’ fitness using values between 0 and 1. Values nearer to 1 depict a mutual relationship, while values closer to 0 indicate a weaker association. The mean absolute error (MAE), which measures the mean absolute distance between predicted and true values, and the mean squared error (MSE), which shows the possibility of considerable mispredictions were also adopted for model validation. Eqs. (1)–(3) show the formula for calculating the $\textrm{R}^{2}$, MSE, and MAE, respectively.

1 $$\begin{aligned}{} & {} \text {R}^{2}=\frac{\sum _{i=1}^{n}\left( X_{i}-X_{m}\right) \left( Y_{i}-Y_{m}\right) }{\sqrt{\left( \sum _{i=1}^{n}\left( X_{i}-X_{m}\right) ^{2}\right) \left( \sum _{i=1}^{n}\left( Y_{i}-Y_{m}\right) ^{2}\right) }} \end{aligned}$$

2 $$\begin{aligned}{} & {} \text {MAE}=\frac{1}{n} \sum _{i=1}^{n}\left| Y_{i}-X_{i}\right| \end{aligned}$$

3 $$\begin{aligned}{} & {} \text {MSE}=\frac{\sum _{i=1}^{n}\left( Y_{i}-X_{i}\right) ^{2}}{n} \end{aligned}$$

where *n* is the total number of data points or instances, $X_{i}$ and $Y_{i}$ are the actual and predicted values, respectively, $X_{m}$ and $Y_{m}$ are the mean of the actual and predicted values, respectively.

### Machine learning techniques

- *Linear regression* is a statistics-based machine learning model used for quantitative analysis and prediction of numerical variables based on correlation, and it is used to determine how well one or more explanatory variables can linearly predict the response variable. For this study, the response variable is the predicted ozone concentration, while the explanatory variables are the meteorological variables.
- *Support vector regression* (SVR) is a supervised learning algorithm for regression, which is versatile, since it fits linear and nonlinear models, thanks to the availability of its special functions, called kernel functions^33^. In this study, the linear kernel was used. It has more flexibility in choosing penalties and loss functions and scales better to large numbers of samples^34^.
- *Decision trees* (DT) is a non-parametric supervised learning method used for classification and regression. Its purpose is to create a model for prediction by learning decision rules from the characteristics of the data^35^. Basically, the decision trees apply a sequence of decisions that often depend on a single variable. These trees divide the input into regions, refining the level of detail at each iteration until reaching the end of the process, also called a leaf node, which provides the expected end label^35^.
- *Random forest* is a machine learning combination algorithm that can perform classification, regression, clustering and variable selection^36^. Is based on the combination of decision trees. Each tree is constructed using a bootstrapped sample of the data. The final class is predicted, and output is resolved based on the number of the decision trees’ vote^36^. For this study, the RandomForestRegressor of scikitlearn was used in python, and the maximum depth of the tree equal to 2.
- *The multilayer perceptron* (MLP) model consists of a set of elementary processing elements called neurons^37^. These units are organized in architecture with three layers: the input, the hidden, and the output layers. The neurons corresponding to one layer are linked to the neurons of the subsequent layer. An important factor in the specification of neural models is the activation function’s choice. These can be non-linear functions as long as they are continuous, bounded, and differentiable. The transfer function of the hidden neurons should be nonlinear while for the output neurons the function could be a linear function or nonlinear functions^37^.

## Results

### Correlation analysis: meteorological variables vs. O$_{3}$

**Figure 2:**
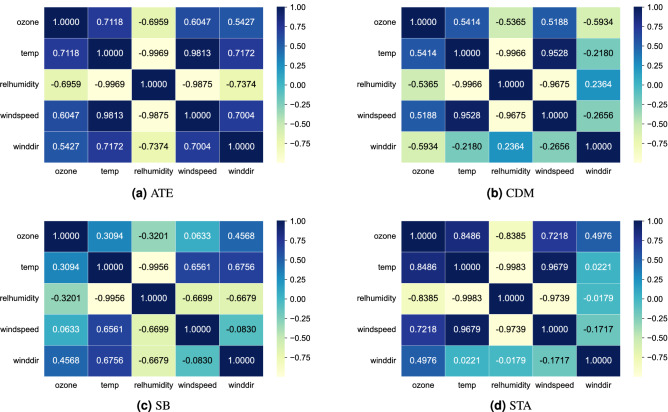
Correlation matrices considering mean between the meteorological variables and the ozone for each monitoring station. This correlational analysis allows evaluating the associations between the variables under study. The reported values oscillate between $-1$ and 1, when there is a negative and positive association, respectively.

**Figure 3:**
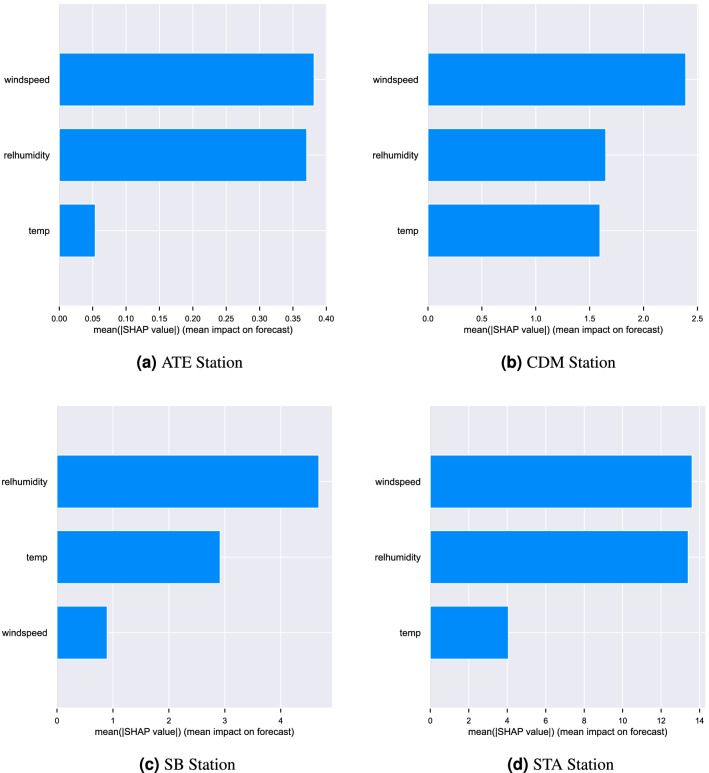
Mean impact of selected variables on ozone. Each variable is observed ordered according to the impact with respect to ozone. In the same way, it is observed that both wind speed and relative humidity have the greatest impact on the model.

Ozone concentration was analyzed with the meteorological variables for the four monitoring stations. Figure 2 shows that the correlation between temperature and ozone for the four monitoring stations ranges between 0.3094 and 0.8486. There is a positive, directly proportional correlation between the two. This aligns with the results of other studies that established a connection between ozone and temperature^38^. Also, mentioned that changes in the intensity of solar radiation lead to large seasonal differences in O$_{3}$ concentrations. High temperatures and ultraviolet radiation accelerate the production of ozone^39^. This directly proportional association between temperature and ozone has an impact on the winter season, that is, the phenomenon that the lower the temperature, the lower the ozone concentration occurs (phenomenon that occurs in Metropolitan Lima). Regarding wind speed and ozone concentration, a strong positive correlation is shown between the four monitoring stations. Both variables are directly proportional in terms of their increase. The correlation indices for the stations range between 0.0633 and 0.7218. A higher level of ozone occurs as the wind speed increases, while the lowest ozone concentration is recorded in the absence of wind^39^, since the effect generated by the meteorological variable on the O$_{3}$ concentration decreases its levels due to the dispersion it generates^6^. Figure 2 also shows a strong negative correlation between relative humidity and ozone. This ranges between $-0.3201$ and $-0.8385$. Low humidity is a suitable climatic condition for photochemical reactions in ozone production^6^. This contaminant is easily soluble in water^40^, which indicates that the presence of precipitation increases the speed at which it dissolves^41^. In addition, Lima is a city with a high relative humidity index^42^, which causes ozone concentrations to decrease in the winter season. Likewise, it is shown that the strongest correlations between climatic variables and ozone concentration occur at the ATE and STA monitoring stations. These areas are more exposed to air pollution, since both districts are located at key points of industrial development, vehicular traffic and fuel combustion^30^. On the other hand, the adoption of five machine learning algorithms was required to determine the reliability of these climatic variables as predictors of the ozone variation trend. In addition, the importance of evaluating the correlation between ozone and climatic variables establishes indicators for future modeling of concentrations of atmospheric pollutants. To observe the average impact of each variable for the prediction of the variable of interest, we used the Shapley Additive Explanations (SHAP) method^43^. The results (Fig. 3) shows that relative wind speed and relative air humidity are the features with higher impact on ozone forecast, that is, the variables most relevant to model’s prediction.

**Figure 4:**
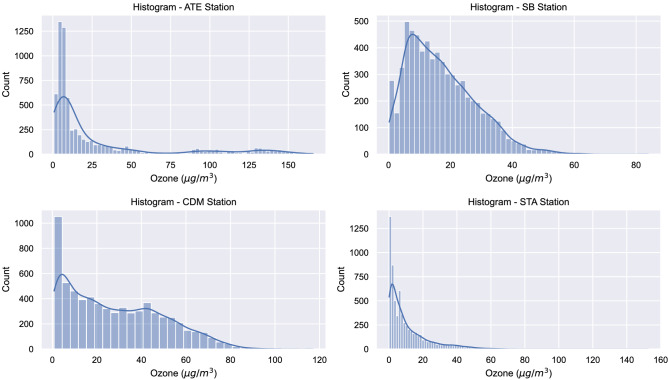
Ozone histogram per each monitoring station. This analysis shows how the ozone behavior is in the different monitoring stations. Likewise, it is complemented with the descriptive analysis, provided by Table 2, where it reports positive asymmetry for all seasons.

**Table 2:** Ozone description by monitoring stations.

	ATE	CDM	SB	STA
Mean	28.36	28.13	17.09	10.56
SD	40.08	21.31	11.05	12.21
Min	0.80	0.80	0.20	0.10
25%	5.50	8.98	8.30	1.80
50%	8.50	24.50	15.10	6.20
75%	29.30	44.03	24.00	14.80
Max	165.80	117.10	83.90	152.60
Skewness	1.89	0.53	0.83	1.94
Kurtosis	2.30	$-0.67$	0.51	5.54

The highest contamination average is in ATE. It also reports the highest pollution indices, presenting a positive asymmetry. Likewise, SB is the one with the lowest contamination index.

### Critical episodes of O$_{3}$

**Figure 5:**
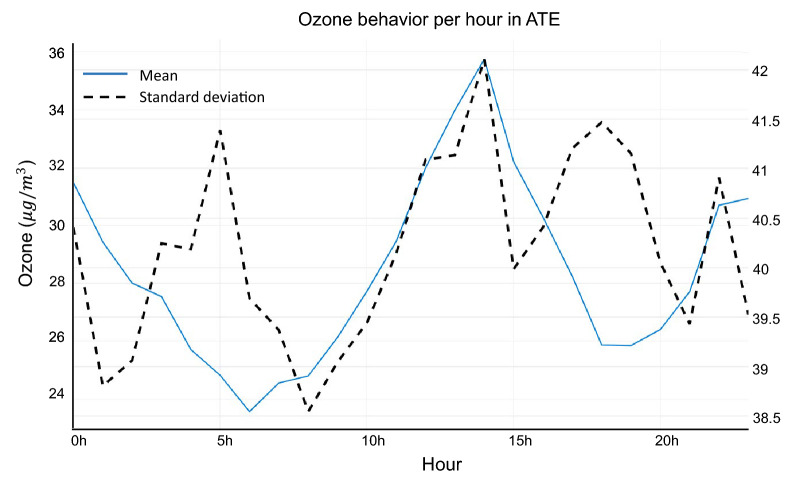
Analysis of average contamination in hourly scale. In this analysis it is observed that the peak hours are 0 h and 14 h. This provides the restriction for the exploratory analysis, reported in Fig. 6.

**Figure 6:**
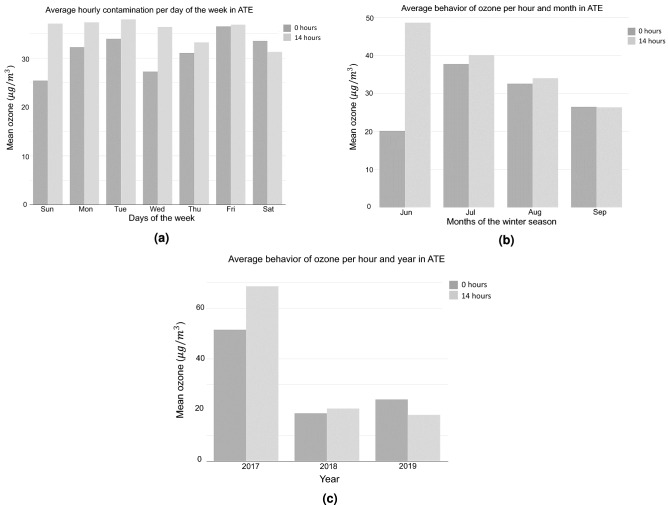
Exploratory analysis per **(a)** days of the week, **(b)** months of the winter season and **(c)** year considering peak hours of contamination in ATE.

We consider critical episodes those values that show an unusually high or low behavior. These data often exhibit excessive kurtosis and/or prominent right tails (see Table 2). Critical ozone episodes were analyzed, contrasting with preliminary work^44^. Previously, histograms were generated to evaluate their behavior (Fig. 4) in the four monitoring stations. Data was used on an hourly scale on all winter days from 2017 to 2019. It should be noted that the ATE station was taken as a reference, because it shows higher levels of pollution since it is considered an industrial and commercial zone^30,31^. Likewise, for greater identification, the behavior of the mean and standard deviation of ozone was reported (see Fig. 5), showing that the pollution peaks are at 00:00 hours and at 14:00 hours. While, to increase the perception of critical episodes and to know their behavior, an average study was carried out on a daily, monthly and annual level (see Fig. 6). The other monitoring stations have the following: CDM (04:00 and 13:00 hours), SB (03:00 and 14:00 hours) and STA (04:00 and 14:00 hours). Regarding the hourly average of pollution per day of the week at the ATE station, a higher index is observed at 2:00 p.m. every day, corresponding to a greater vehicular, commercial and industrial flow^27^, except Thursday and Saturday (pollution declines). On the other hand, in the other monitoring stations there is a greater concentration on Friday, Saturday and Sunday, at 1:00 p.m. (CDM) and 2:00 p.m. (SB and STA). This phenomenon has already been analyzed, being called the “weekend effect”, and is characterized by a high growth of O$_{3}$ in the urban areas compared to working days^45^. In relation to the monthly behavior (Fig. 6), this presents a moderate increase in the month of June, and low in the months of July and August, in the three years. This is due to the fact that episodes of high pollution are not only affected by precursor gases in the environment^19^, but also by the meteorological conditions of the area, especially when knowing the high rate of dispersion that ozone has compared to with other contaminants^8^. In Lima, in the month of June, there are conglomerations of air masses that originate in the Pacific Ocean, with a complete route from the city to the eastern part, an area in which it stops, suspending the ozone and decreasing the quality from air; this being the point where the ATE district is located^27^. In the other monitoring stations, the following results were given: CDM (July), SB (August) and STA (July). On the other hand, the direction of the wind presents a greater predominance towards the south-west in the monitoring stations, with a speed that oscillates between 0 and 4.3 m/s. Regarding the analysis by year, it is possible to visualize the pronounced variation of critical episodes recorded at the ATE station, with 2017 being the year most affected by pollution. This is due to the fact that the increase in the industry in that year was transcendental, thus generating the emission of precursors such as: NO, CO and VOC. Even reports from the municipality mention that on several occasions, this district exceeded the breaking point imposed by current laws^27^, and this is reflected in Table 2, where the maximum values reached 165 $\upmu$g/m$^{3}$ this result being 65% higher than the norm. In the other monitoring stations, the following result was given: CDM (2019), SB (2017) and STA (2017).

### Models’ performance

**Figure 7:**
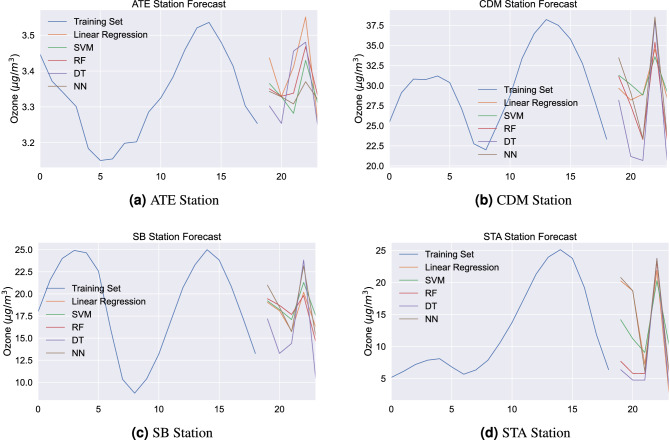
Forecast results plot. Observed data for ozone (training set) and forecast results for applied models: linear regression, support vector machine, random forest, decision tree, and neural network.

The results described in Table 3 consider three precision metrics: multiple determination coefficient, mean squared error and mean absolute error. The coefficient of determination has a variation between 0.4190 and 0.9933, showing that all models are able to explain the average variation in the ozone level. It should be noted that five steps ahead were considered as the forecast horizon and the data were grouped according to the average at each time during the entire period collected. The MAE and MSE metrics show that the models have a good predictive capacity for all monitoring stations that were investigated. The MSE and MAE of all the algorithms give a low value, which shows the predictive performance’s accuracy. A comparative study was proposed by^38^, with the result that the best models to model ozone in Malaysia are: random forest, linear regression, support vector regression and decision tree regression. The study did not investigate the multilayer perceptron model, but the results found are consistent with what we found in this study. Furthermore, the variation of R$^2$ was 0.216 and 0.970. The Fig. 7 shows the forecast results for models applied in this study. It is possible to observe that the models have results with similar behaviors - even at different levels. The results are important to present the predictive capacity of the models for the analyzed variable of interest. Regarding the use of support vector machine, the study analyzed the use of a linear kernel and an RBF type kernel. Results were better through a linear kernel. For Random Forest, simulations with a max depth between 2 and 100 were considered, with the best result obtained with 2. And for the MLP model, a log-sigmoid function was considered. The comparison between the applied machine learning models shows that the linear regression model obtained the best prediction results for CDM and SB stations. For the ATE station, the SVM model was the best for the MAE metric and the linear regression model was the best for the R$^2$ and MSE metrics. And finally, the SVM model was the best for the STA station. Thus, it is shown that the best models were the linear regression model and SVM. The results found by the models, even with forecast variations, are shown as valid behavior variables to analyze and run at the monitoring stations.

**Table 3:** Result of the R$^2$, MAE and MSE metrics applied to the forecast results for the analyzed models.

Station/model	R$^2$	MAE	MSE
**ATE station**			
LR	0.9923	0.0724	0.0058
SVM	0.9913	0.0643	0.0065
DT	0.9478	0.1648	0.0392
RF	0.8753	0.2373	0.0937
MLP	0.906	0.2481	0.0706
**CDM station**			
LR	0.9892	0.0486	0.0036
SVM	0.9844	0.0702	0.0052
DT	0.873	0.1915	0.0424
RF	0.7576	0.2286	0.0809
MLP	0.9246	0.1352	0.0251
**SB station**			
LR	0.9849	0.0847	0.0087
SVM	0.9814	0.0923	0.0107
DT	0.8728	0.199	0.0729
RF	0.9699	0.096	0.0172
MLP	0.419	0.5149	0.3332
**STA station**			
LR	0.9909	0.0758	0.006
SVM	0.9933	0.0501	0.0044
DT	0.8349	0.2497	0.1081
RF	0.8203	0.2842	0.1176
MLP	0.9452	0.1783	0.0359

The results are separated by station (ATE, CDM, SB and STA).

## Discussion

The impacts of ozone on air quality in metropolitan Lima were modeled using machine learning techniques. The concentration of O$_{3}$ in the metropolis presents critical levels, mainly in ATE, compared to the other monitoring stations (CDM, SBJ, STA) on an hourly scale. Typically, low temperatures, excess relative humidity and wind speed are influencing factors for records of low O$_{3}$ levels. In a study in Beijing^46^ was mentioned that low humidity is a suitable climatic condition for photochemical reactions in ozone production. Metropolitan Lima is a city with a high relative humidity index and more in the winter season^16^. Therefore, it acts as an important factor to decrease the increase in ozone concentration, since it is associated with precipitation and solubility in water. The use of artificial intelligence models (such as machine learning methods) is important to propose new approaches for definition of environmental public policies. In this case, economic agents can benefit from more accurate results and improve their decision-making process and monitoring of nature phenomena. And is important to investigate the use of interpretability methods (as we make in this study). Among the climatic factors addressed in this study, temperature became an important variable since it has a strong relationship with O$_{3}$. In the study by Ocak and Turalioglu^47^, found that they had high levels of ozone during warm periods; this phenomenon is complemented by the case of Metropolitan Lima during the cold period, since the values of ozone concentrations decline. The results obtained in our study support that, in the winter season, Lima presents O$_{3}$ values on an hourly scale below 100 $\mu$g/m$^3$ according to the ECA for air^18^. This is because critical episodes occur on some weekdays during peak hours. The main cause is vehicular traffic, since transport generates this pollutant^48^. On the other hand, temperature differences result in the movement of air masses from lower to higher temperatures, causing local winds that are recorded on the coast, transporting the pollutant in a south-southeast direction^49^. In this sense, the high levels of ozone can be mitigated by adopting measures such as the substitution of the classic fuel for gaseous ones (NGV, LPG) and biofuels^50^. With all this, through Table 4, different comparisons of approaches between Metropolitan Lima (Peru) and China are summarized, providing a broader spectrum regarding ozone.

**Table 4:** Behavior of ozone evaluated both in China and in Peru (Metopolitan Lima).

References	China	Lima
China^8^ Lima^16^	O$_{3}$ concentrations above current air quality standards were identified, influenced by the Northwest Pacific typhoon, a frequent weather activity in hot seasons	In June there are conglomerates of air masses that originate in the Pacific Ocean, suspending ozone and reducing air quality in surrounding areas
China^39,51^, Lima$^{\text {This study}}$	Hourly O$_{3}$ concentrations show that levels gradually increased after 08:00 h (33,8 $\upmu$g/m$^{3}$), peaked at 16:00 h (96.7 $\upmu$g/m$^{3}$), and then decreased. Furthermore, O$_{3}$ concentrations were highest in summer and lowest in winter	O$_{3}$ concentrations evaluated in the winter season report 0.10 $\upmu$g/m$^{3}$ at 02:00 h, while at 18:00 h a high concentration of 165.8 $\upmu$g/m$^{3}$ is reported
China^51^, Lima$^{\text {This study}}$	Of the 13 cities evaluated, the lowest O$_{3}$ concentration was concentrated in Chengde (82.7 $\upmu$g/m$^{3}$), while the highest was in Hengshui (98.4 $\upmu$g/m$^{3}$)	Likewise, of the stations observed in Lima, the lowest O$_{3}$ levels are concentrated in the STA district (0.10 $\upmu$g/m$^{3}$), while the highest are found in ATE (165.8 $\upmu$g/m$^{3}$)

## Conclusions

This study modeled and analyzed the concentration of ozone on air quality in Metropolitan Lima during the winter season. Correlation analysis and five machine learning techniques were used to obtain the relationship between meteorological variables and ozone, highlighting linear regression and support vector machine as techniques that showed better predictive capacity. In parallel, the correlation analysis shows a strong positive relationship between temperature and ozone. Also, there is a strong positive relationship between wind speed and ozone. While, between relative humidity and ozone there is a strong negative relationship (inversely proportional) in the four monitoring stations, indicating their high exposure to the pollutant. From this, it is determined that the air quality of urbanized areas is significantly associated with fluctuations in meteorological factors. This problem is generated by anthropogenic activities. Taking into account climatic variations, this study provides a solid basis for interventions in the most vulnerable areas. In addition, it opens the gap for future analysis and understanding of the behavior of meteorological variables and ozone. Likewise, this research presents great modeling potential, through machine learning algorithms for simulations of the urban variability of ozone in the Lima metropolitan region, which will serve as a reference for future ozone modeling applications. However, further studies may be needed to improve the fit by incorporating more input variables that have not yet been investigated due to lack of data and information. Alternatively, in the context of COVID-19, it would also be interesting to evaluate and model the behavior of ozone, using statistical techniques such as multiple linear regression, use of three-dimensional logarithms and principal component analysis, under the influence of meteorological variables in the warm and cold season, similar to what was developed for PM$_{10}$^52^, obtaining important reports for decision-making in environmental management.

For their part, subsequent studies can be extended to additional contaminants with classification approaches under the standards established by the country. On the other hand, the partially varying coefficient model approach with heavy tails could be addressed for ozone, since it was successfully addressed with PM$_{10}$^53^. Also, evaluate through time series, the trends of meteorological variables and ozone, in order to broaden the understanding of the correlation between the variation of climatic variables and the variation of ozone concentration.

## Data Availability

The datasets analysed during the current study are available in the *Servicio Nacional de Meteorología e Hidrología del Perú* repository: https://www.senamhi.gob.pe/?p=calidad-del-aire.
